# Affections of the Cervix Uteri

**Published:** 1855-07

**Authors:** Luther Parks

**Affiliations:** Boston


*The Question of the frequency of Inflammatory Affections of the Cervix Uteri; and also that of their Pathological Value*, By LUTHER PARKS, Jr., M. D., Boston.

*Frequency.*—Inflammatory affections of the cervix uteri are thought by Dr. Robert Lee to be rare. The views of this author are shared, to a greater or less extent, by Dr. Ashwell and some others.

The grounds of disbelief in the frequency of these affections are derived from results of autopsies and of observation in the living subject.

Dr. Lee states that a large number of autopsies were made at the St. Marylebone Infirmary, and at St. George's Hospital, in which inflammation and congestion were found in but a small proportion, and ulceration of a doubtful character in but a minute proportion of cases. One hundred and eighty uteri were examined at St. George's Hospital by Mr. Gray, who found redness, slight abrasions and granulations, sometimes, but not frequently—ulcerations never, except of a specific character. "In a considerable number of cases in which ulceration had been affirmed by others to exist, after repeated and deliberate examinations with the speculum," Mr. Gray ascertained that "ulceration did not exist in the os and cervix uteri—nor disease of any kind."

The result of Dr. Ashwell's observation through many years' experience was "decidedly opposed to the views of uterine pathology which had of late years been so industriously propounded." Out of a thousand (1026) cases of actual uterine disease seen by himself and pupils, only twenty-five were found to be instances of inflammation of the uterine neck.

Dr. Lee also states that he has frequently used the speculum, but has never seen *ulceration* of the os and cervix uteri, unless of a specific, and especially of a scrofulous* and cancerous character.

In opposition to the foregoing statements are opposed the experience of Drs. Bennet, Murphy and others who have been *in the habit of using the speculum.*

Dr. Bennet explains the results of *post-mortem* examinations at the Marylebone Infirmary, by recalling on the one hand, "the well-known fact" that "the most eminent pathologists often passed over important lesions without observing them, until their attention had been directed to them," and declaring, on the other hand, that when the above-mentioned researches were made, "the practical knowledge of the inflammatory lesions of the cervix uteri did not exist in the profession." Moreover, remembering that the females in question died from general disease without the existence of any uterine ailment having been suspected, the discovery of such lesions, even in a limited number of cases, is, of itself, he thinks, a clear proof of the not unfrequent existence of the disease.

In reference to the cases quoted by Dr. Ashwell, he opposes his own observations, from which it results that out of three hundred cases presenting uterine symptoms, there were two hundred and forty-three of inflammation, and two hundred and twenty-two of ulceration. The discrepancy he explains by supposing that a speculum examination not being considered, at the time, by Dr. Ashwell to be warranted, the real nature of the complaint was not recognized in the majority of his cases.

* It is worthy of notice that Dr. Lee here acknowledges having seen *ulceration*—though called by him scrofulous.

Dr. Lee, also, we may add, objects strongly to the employment of the speculum, save in exceptional cases, relying upon the dignital examination, which has been shown by Dr. Bennet to be unreliable.†

In confirmation of Dr. Bennet's position, Dr. Murphy‡ states that he has been in the daily habit of seeing cases of inflammation of the uterine neck, in all the stages of its progress. He has seen hundreds of cases of uterine disease, and declares that seven-tenths were cases of inflammation of the cervix uteri.

The question of ulceration has been, also, further discussed upon a somewhat different basis. The fact of certain appearances being taken as a postulate, the question is considered, "do these appearances denote ulceration?" Dr. Snow Beck replies in the negative, claiming that only abrasion has been proved to exist. Dr. Lee acknowledges having seen *excoriations* or *granulations* upon an intensely-red base (an important admission we may remark by the way;) and the writings of Dr. Snow Beck (also until recently, those of Dr. Tyler Smith) tend to the point that these are in reality the appearances described by Dr. Bennet as ulcerations; that there is in them no solution of continuity, and, consequently, that they do not deserve the title of ulcerations.

Dr. Bennet, on the other hand, quotes authorities to sustain his use of terms, and states that in the appearances he describes, the granulations may be "large, fungous, livid, and bleeding at the slightest touch."

Dr. Murphy also says that in the cases he alludes to (as cases of ulceration) there should be found "a circumscribed inflammatory surface secreting pus;" and implies the question—"what is the inference in regard to such an appearance?"

An answer to this question may be found in the results of the brilliant researches upon the subject lately conducted by Dr. Tyler Smith. Dr. Smith states that in the course of his microscpical investigations of the cervix uteri, the basement membrane, with its superimposed epithelial layer, was found to cover numerous villi or papillæ.

Now, Dr. Smith states that with the assistance of Dr. Hassall he has examined many cases of "abrasion or superficial ulceration." In a portion of these cases there was merely a loss of epithelium, and he declares that in analagous states in the living subject, the mucous surface is of an intensely red color from the presence of the naked villi, with their vascular loops, and conveys the "velvety" sensation which has been described as indicative of ulceration; the villi, in this condition, standing out like the "pile of velvet," and be-

† M. Dupuytren also observes that "mucous ulceration" of the cervix uteri may be easily overlooked if we proceed no further than an examination with the finger.

‡ Of the University College, London.

ing in some cases considerably enlarged. But, in other cases there is not merely loss of the dense epithelium, but the *villi*, both of the external surface of the os uteri, and of the mucous surface within the labia uteri, *are destroyed in patches.* In that condition of the os uteri, which, upon examination after death would be pronounced *undoubted superficial ulceration*, the state which generally obtains is partial or entire loss of the epithelial layer in circumscribed patches, and here and there *the loss or partial destruction of the villi.*" * * * * "Sometimes small circumscribed *ulcers* were seen, in which the denuded or partially denuded villi are found surrounding the edge of the small ulcer, *the area of the ulcer itself being bare of villi*, and the ragged *debris* of villi, and their vascular loops, appearing at the bottom of the *ulcer.*"

Again, he says, by far the most common lesion is "*epithelial abrasion*;" but there is a second grade in which "the *villi* and occasionally *the base from which they spring* are effected from a superficial ulcerative process, which may be designated as *villous abrasion*, *erosion* or *ulceration.*"

Finnally we quote the following words of Dr. Smith. "In one case which I examined after death, not only the villi, but *portions of the lower rugæ* in the glandular portion of the cervix were eaten away."

Now, what shall we conclude but that there is such a thing as *ulceration* of the cervix uteri, though less frequent than an antecedent stage of the inflammatory process—viz., *abrasion.* The distinction between these two states we believe to be not always borne in mind, and to be not always easy. It is, however, not of the highest practical consequence. For, although *other things being equal*, a less degree of disease will cause less suffering than a greater, yet in practice the "other things" are so far from being actually equal in different cases of female complaints, that I had almost declared the intensity of the symptoms to bear a correspondence less close to the intensity of the disease, than to the idosyncracy of the patient.

To revert to the question of the absolute frequency of ulcerative appearances, we have seen that Dr. Bennet found them in two hundred and twenty-two out of three hundred cases presenting uterine symptoms. We will add that Mr. Whitehead, as the result of very extensive observation, found them to be a very frequent occurrence in the females of the city of Manchester. But, finally, last, but not least, we have the statement of Dr. West, of London, in a work lately written to disprove the pathological importance of the ulceration of the os uteri, that of sixty-two uteri taken from patients who died in the medical wards of St. Bartholomew's Hospital, of *other than uterine disease*, seventeen were affected with ulceration of the os (a most striking fact under the circumstances;) and that out of two hundred and sixty-eight cases in which the speculum was used in the living subject, the os uteri was found to be the seat of ulceration in one hundred and twenty-five.

We consider, therefore, the frequency of ulcerative appearances, at least as well as of other forms of inflammation, fully proved.

We have now a word to say upon the *pathological value of inflammatory affections of the uterus.* From the general tone and drift of Dr. West's paper, already referred to, and of the remarks of those who, as reviewers, have eulogized it, one might be led to infer that not alone *ulceration* was under consideration, but that *inflammatory diseases* generally had been the object of his investigations. Dr. West dogmatizes as though he had shown that the diseases requiring ocular inspection of the womb must be comparatively rare, so much so that the employment of the speculum must therefore seldom be requisite. He combats the modern views of uterine pathology which locate, so often, in the *uterus* the source of uterine symptoms, compassionating its supporters as mistaken enthusiasts, and, while acknowledging that ulceration of the os uteri is not absolutely unimportant, intimates the more frequent dependence of uterine ailments on constitutional causes. These conclusions would seem, from his course of remark, to flow as deductions from his observation of the numerous cases examined by the speculum. His results are given in the form of tables, and, so far as they go, are admirably presented. But, an examination of these tables, and even his own summary of results, when he distinctly presents it, shows that no such conclusions as the above are warranted by his statistics. The only inferences deducible affect, not inflammatory affections generally, but only *ulceration*—and that, simply in comparison with other uterine affections, other forms of inflammation, for all that appears to the contrary, among the number.

Out of 1226 cases, in 268 the symptoms appeared to Dr. West to justify the use of the speculum. From these 268 cases (which he divides into two classes—those in which the os uteri was found to be the seat of ulceration, and those which showed no sign of that condition,) he chiefly draws his conclusions, though for some purposes, he properly compares them with other cases.

Some of the most important results which he deduces from the above cases are, that, while "menstruation was found to be oftener excessive, leucorrhœa to be more frequently profuse, in cases where the os uteri was ulcerated," (and in like manner, he says "the existence of that condition seems to be accompanied by pain diffused generally over the whole pelvic region more frequently when the os uteri is ulcerated than when ulceration is absent;) and, while "the symptoms"—identical in character with the two classes of cases"

—"seem to present a slightly increased degree of intensity in those instances in which ulceration of the os uteri existed—yet, "uterine pain, menstrual disorder, and leucorrhœal discharges—the symptoms ordinarily attributed to ulceration of the os uteri—are met with independently of that condition almost as often as in connection with it."

Now, I would briefly ask, what is there in these conclusions, which, if they had been enunciated by Dr. Bennet himself would be inconsistent with his teachings? Is it to *ulceration* that his catalogue of *symptoms*, strictly speaking, is referred? Does he not constantly consider *inflammation* and *ulceration* together, save in speaking of those local changes—the consequences of ulceration (as, for instance, hypertrophy) which he sees fit to term the *anatomical* symptoms of the latter state? Nowhere, so far as I know, does Dr. Bennet or any other observer assign to ulceration the predominance above intimated over at least other inflammatory affections of the cervix uteri.

Let us now, by way of further example, analyze some of the results of Dr. West's first table—that prepared with reference to the relation between ulceration and *sterility*.

In 980 healthy women delivered by pupils of St. Bartholomew's, the average number of children to each marriage was 4.17.

In 980 patients with uterine symptoms, examined or not with the speculum, the average number of children to each fruitful marriage was 2.7.

In 125 patients* *with* uterine symptoms, examined with the speculum, in whom no ulceration was found, the average number of children to each fruitful marriage was 3.3 ; In 117 patients with uterine symptoms, and with ulceration, as shown by the speculum, the average number of children to each fruitful marriage was 3.5.

In the total number (980) of patients with uterine symptoms,† the proportion of sterile marriages was 1 in 8.5. In those of the above who were examined with the speculum (125 in number) and found not to present ulceration, the proportion of sterile marriages was 1 in 5.2. In the remainder (117) in whom the speculum showed ulceration, the proportion of sterile marriages was 1 in 7.3. *But no statistics were obtained of the proportion of sterile marriages in healthy women, or women without uterine symptoms.*

Now, as says Dr. West himself, "we cannot but be struck with the great diminution in fecundity in those women who were suffering from ailments of the generative system;" and we may add that to those who will believe that in the absence of proof to the con-

* It should be noted that no statement is given as to whether or not other forms of inflammatory affections than ulceration existed in these 125 cases.

† The clause capable of affording statistics in this connection should be inserted.

trary, the larger portion of these cases was made up, wholly or in part, of inflammation (their belief being founded upon the statistics of West himself as well as upon those of Bennet,) the above facts will be confirmatory of the influence of inflammation upon conception.

"This result, however," Dr. West remarks, "instead of being more marked in cases of ulceration of the os uteri than in those where no such condition existed, appears in reality to be less so."* In reply to this proposition I would ask, who has alleged the greater or less influence of ulceration in this respect? Has any one made ulceration the cause of sterility, instead of placing it *among* the causes of that state? Has, in fact, ulceration been dwelt upon as (more than other forms and terminations of inflammation) a specially active cause of sterility? Certainly it has not been thus dealt with by Mr. Whitehead or Dr. Bennet.

Mr. Whitehead enumerates the causes of sterility thus—"diffuse chronic endo-enteritis; morbid states of the uterine and vaginal secretions—their deleterious effect upon the spermatic animalcules." "Chronic endo-enteritis," he says, or what may be called irritable uterus [*not ulceration*,] is in fact one of the most frequent causes of sterility."

In the 9 cases of sterility reported by Mr. Whitehead, the attending lesions mentioned are sanious leucorrhœa and infirm health; sanio-purulent leucorrhœa, endo-enteritis, dysmenorrhœa, secondary syphilis, procidentia uteri, profuse leucorrhœa, ulceration and erosion. In none of the above cases is ulceration set down as the sole diseased condition, and in four only is it mentioned as occurring at all.

I rise, in fine, from Dr. West's work with the impression that while the large proportion of speculum examinations which revealed ulceration by Dr. West are of great value as confirming the experience of Dr. Bennet and others, in relation to the frequency of ulcerative appearances (the observations being made by one who would not be likely to see the lesion in question, *where it did not exist*,) the former observer's statistics do not invalidate any of Dr. Bennet's positions, unless it be, perhaps, to a certain extent, the influence of ulceration upon hypertrophy.

In conclusion, I would remark in relation to the pathological value of inflammatory diseases of the cervix uteri generally, that while the causes to which female ailments were formerly assigned, were vague and unsatisfactory, and their treatment inefficient, the modern uterine pathology, in ascribing those ailments in a large degree to uterine inflammation, is, to some minds at least, tangible

* In justice to Dr. West, it should be stated here that he observes that "the number of facts from which this table is constructed are too few to justify any such inference" as that sterility is less likely to occur where there is ulceration than in other cases.

and rational, and the modern treatment productive of the removal of the symptoms. In a word, females present themselves with certain abnormal appearances at the cervix uteri, and with certain symptoms. A certain course of treatment is adopted, *unlike that formerly employed*, and as a general thing the abnormal appearances are removed, and *unlike what formerly happened*, the symptoms take their departure.—[*Bos. Med. and Sur. Journal.*

